# The w-eCura system is a novel indicator of the need for salvage surgery after non-curable endoscopic submucosal dissection in early gastric cancer: A retrospective case-controlled study

**DOI:** 10.1371/journal.pone.0348876

**Published:** 2026-05-19

**Authors:** Yangyang Chen, Fengqin Fu, Junyan Lin, Xiaoling Zheng

**Affiliations:** 1 Digestive Endoscopy Department, Shengli Clinical Medical College of Fujian Medical University, Fuzhou, Fujian, China; 2 Digestive Endoscopy Department, Fuzhou University Affiliated Provincial Hospital, Fuzhou, Fujian, China; 3 Digestive Endoscopy Department, Fujian Provincial Hospital, Fuzhou, Fujian, China; 4 Department of Gastroenterology, Zhangzhou Municipal Hospital Affiliated to Fujian Medical University, Zhangzhou, Fujian, China; Universitá Sapienza di Roma, ITALY

## Abstract

**Objective:**

This study sought to evaluate the effectiveness of the w-eCura system in determining the necessity of salvage surgery after non-radical endoscopic submucosal dissection (ESD) for early gastric cancer (EGC) and to compare its efficacy with the eCura system.

**Methods:**

This retrospective case-control study was conducted at Fujian Provincial Hospital and included 290 patients with EGC who underwent ESD between January 2011 and July 2024. All patients were confirmed with non-radical resection upon pathological evaluation. Various clinicopathological characteristics such as gender, age, lesion location, size, Paris classification, histological type, depth of invasion, ulceration, lymphatic and venous invasion, and margin status were documented. Subsequent salvage procedures, lymph node involvement, and recurrence were monitored. Risk stratification was performed using the w-eCura and eCura systems, and the Cox proportional hazards model was employed to identify factors influencing positive cancer residue status (CRS). Survival analysis was conducted using Kaplan-Meier curves, and the predictive performance of the two models was compared using receiver operating characteristic (ROC) curves.

**Results:**

The study included patients with a mean age of 64.42 ± 9.35 years and a median maximum tumor diameter of 4.50 cm, who were monitored for a median duration of 32 (range 15–54) months. Factors such as w-eCura grade, horizontal resection margin, depth of invasion, lymphatic invasion, and venous invasion exhibited significant associations with CRS-positive classification (P < .05). A higher w-eCura grade was linked to an elevated risk of CRS-positive classification. Utilizing Cox proportional hazard models, we identified that lesion location (The middle 1/3 stomach), w-eCura grade (high and intermediate), and infiltration depth (SM2 and SM3) independently influenced CRS-positive classification. Kaplan-Meier survival analysis demonstrated a significant correlation between both eCura and w-eCura grades and survival time (Logrank P < .001). Notably, the w-eCura classification displayed enhanced sensitivity in detecting high-risk patients promptly. ROC curve analysis revealed that the w-eCura model exhibited an area under the curve (AUC) of 0.82, surpassing the eCura model’s AUC of 0.79 and underscoring the superior predictive accuracy of the w-eCura model for CRS-positive classification. Within the intermediate-risk w-eCura subgroup, positive vertical resection margin and SM3 infiltration depth emerged as independent factors influencing CRS-positive classification.

**Conclusion:**

The w-eCura system demonstrated superiority as a tool for assessing the necessity of salvage surgery following non-curative ESD in EGC compared to the eCura system. For patients presenting intermediate-risk, the w-eCura system in conjunction with positive vertical resection margin or SM3 depth of invasion, additional salvage surgery is advised for consideration.

## Introduction

Gastric cancer is a prevalent malignant tumor posing a significant threat to human life and health, ranking fifth for morbidity and mortality globally [1]. It is categorized into early gastric cancer (EGC) and advanced gastric cancer based on the depth of invasion of cancer cells. EGC is defined as lesions confined to the mucosa or submucosa without infiltration into the musculi propria, irrespective of lymph node involvement [2]. Presently, endoscopic submucosal dissection (ESD) has emerged as the primary treatment modality for EGC patients. However, some individuals undergoing ESD are subsequently deemed to have non-curative resections, necessitating further surgical intervention [2–4]. Among patients who undergo radical surgery following non-curative ESD, the incidence of lymph node metastasis (LNM) ranges from 5% to 10% [5–7]. Consequently, universal application of this treatment approach may lead to unnecessary surgical procedures in over 90% of patients. Therefore, advocating for radical surgery for all individuals post non-curative ESD may be overly aggressive and unwarranted.

The risk factors for LNM and local cancer residual/recurrence following non-curative ESD for EGC remain incompletely understood. In 2017, Hatta et al. introduced the eCura system [8], a 7-point risk scoring scheme based on five clinical factors (lymphatic vessel invasion, vascular invasion, tumor size, invasion depth, and vertical margin) to categorize patients with non-curative ESD resections into either a low, intermediate, or high-risk group, aiding in prognostic stratification. In 2024, Morais R proposed a modification to the eCura system [9], creating the w-eCura system tailored for Western populations. While the w-eCura system has been validated in Western cohorts, its applicability to the Chinese population and its comparative effectiveness against the eCura system in this demographic remain unknown. This study aimed to assess the utility of the w-eCura system in Chinese patients with non-curative resections of EGC after ESD, evaluating its strengths and limitations relative to the eCura system with the goal of informing treatment strategies for this patient population in China.

## Patients and methods

### Study population

This retrospective, case-controlled study was conducted at a single center. Patients underwent ESD treatment for EGC at the Digestive Endoscopy Center of Fujian Provincial Hospital between January 2011 and July 2024. Post-ESD pathology assessment followed the Japanese Gastric Cancer Treatment Guidelines 2018 (5th edition). Inclusion criteria entailed EGC confirmed through postoperative pathology meeting non-curative resection criteria. Exclusion criteria included malignant tumors involving other organs, presence of serious systemic diseases, and incomplete clinical records. A total of 290 patients with non-curative resection of EGC via ESD were retrospectively analyzed. The study adhered to the Declaration of Helsinki and received approval from the Institutional Review Board of Fujian Provincial Hospital (No. K2024-09–043). The patient data was accessed for research purposes on 30/01/2025. Given the retrospective nature of the study, patient data were utilized without explicit informed consent.

### Data collection

Pathological and clinical outcomes of 290 enrolled patients were retrospectively analyzed:

(1) Clinical characteristics included the patient’s gender, age, lesion location, size, and Paris morphological classification. (2) Pathological aspects encompass the completeness of lesion removal via ESD, histological type, depth of invasion, presence of ulceration, lymphatic and venous invasion, and status of resection margins (horizontal and vertical). (3) Follow-up data included details of salvage surgery course, LNM, residual or recurrent disease, and duration of follow-up.

Groups: Cancer residue status (CRS) was characterized by the presence of LNM, residue, or recurrence. Patients were categorized into CRS-positive and CRS-negative groups based on the outcomes of ESD follow-up and salvage surgery course.

### Surgical technique

ESD: In this study, all patients underwent ESD treatment. The ESD treatment process involved several key steps as illustrated: (1) Lesion boundary delineation under endoscopy; (2) Circumferential marking and submucosal injection using a Dual knife to mark approximately 5 mm outside the lesion boundary with a 5 mm interval between marks, followed by submucosal injection with a solution of sodium hyaluronate, methylene blue, and normal saline to facilitate submucosal separation from the muscularis propria and full lesion elevation; (3) Circular incision of the mucosa around 5 mm outside the lesion marking point; (4) Submucosal dissection from the edge until complete lesion dissection; (5) Hemostasis with electrocoagulation using electrocoagulation forceps; and (6) Specimen preparation involving flattening of the specimen with a stainless steel fine needle, placement on a foam plate with the mucosa facing upward, marking the oral and anal sides, immersion of specimen in 10% formaldehyde solution, and evaluation by the Pathology Department.

Radical surgery: Based on the post-surgical pathological findings and tumor size and location, the surgical approach and type were determined among standard radical distal gastrectomy, proximal gastrectomy, or total gastrectomy. All patients underwent standard D2 lymph node dissection following the guidelines outlined in the 2018 Gastric Cancer Treatment Guidelines (5th edition) set forth by the Japan Gastric Cancer Society [10].

### Follow-up

Esophagogastroduodenoscopy (EGD) was conducted at 3, 6, and 12 months post-ESD, with subsequent annual EGD and abdominal computed tomography (CT) recommended for detection of tumor residue or recurrence. Patients with residual or recurrent tumors were advised to undergo endoscopic therapy or further surgery as deemed necessary. Following salvage surgery, EGD and CT were performed at 6 months post-operatively, followed by annual EGD and abdominal CT scans. In the event of relapse, additional treatment was advised.

### Relevant definitions

The lesions were categorized based on their location within the stomach: one-third in the upper stomach (cardia, fundus), one-third in the middle stomach (body), and one-third in the lower stomach (antrum). Lesion size was assessed using pathological specimens to measure both length and diameter.

In 2005, the endoscopic classification of EGC, as per the Paris classification system [11], included three main morphological types: elevated (0-Ⅰ), flat (0-Ⅱ), and sunken (0-Ⅲ). The elevated type (0-Ⅰ) was further categorized into sessile (0-Ⅰs) and pedunculated (0-Ⅰp) subtypes. The flat type (0-Ⅱ) was comprised three subtypes based on lesion characteristics: superficial uplift (0-Ⅱa), superficial flat (0-Ⅱb), and superficial concave (0-Ⅱc). The differentiation between type 0-Ⅰand 0-Ⅱa was determined by an uplift height of 2.5 mm, while the distinction between type 0-Ⅲ and 0-Ⅱc was based on a depression depth of 1.2 mm. Additionally, combinations such as 0-Ⅱc + Ⅱa, 0-Ⅱa + Ⅱc, 0-Ⅲ + Ⅱc, or 0-Ⅱc + Ⅲ are referred to as mixed types.

According to the WHO and Lauren classification systems [12,13], tubular adenocarcinoma and papillary adenocarcinoma are considered differentiated types, whereas poorly differentiated adenocarcinoma, signet-ring cell carcinoma, and mucinous adenocarcinoma are classified as undifferentiated types. Tumors exhibiting characteristics of both differentiated and undifferentiated types are categorized as mixed types. Submucosal carcinoma is defined as carcinoma that invades the submucosa without penetrating the muscularis propria. Submucosal infiltration depths are further classified as follows: infiltration into the upper 1/3 of the submucosa (≤500 μm) is designated as SM1, infiltration into the middle 1/3 of the submucosa (＞500 μm ～ ≤1000 μm) as SM2, and infiltration into the lower 1/3 of the submucosa (＞1000 μm) as SM3.

The histopathological assessment determines the manifestations of ulcers, encompassing both ulcers and ulcer scars. Lymphatic vascular invasion is initially identified through hematoxylin and eosin staining, with immunohistochemistry serving as a supplementary method to confirm ambiguous results.

Complete resection was characterized by the complete removal of the lesion in a single specimen. A positive margin was identified when residual cancer cells were observed at the horizontal or vertical margins of the specimen. Recurrence encompassed local recurrence or distant metastasis to lymph nodes or other organs following treatments such as ESD or surgical intervention for EGC. Patients with positive lymph node metastases were those with cancerous lymph nodes post non-curative resection subsequent to ESD, followed by standard radical surgery. Residual disease denoted the persistence of a tumor at the original site post-ESD.

Per established criteria [4], curative resection is characterized by complete excision with negative margins (both horizontal and vertical) and absence of lymphatic invasion. This includes: (1) predominantly differentiated intramucosal carcinoma without ulcers; (2) intramucosal carcinoma ≤3 cm in size, predominantly differentiated, with ulcers; (3) predominantly undifferentiated intramucosal carcinoma ≤2 cm in size without ulcers; (4) submucosal carcinoma ≤3 cm in size, predominantly differentiated, with infiltration limited to SM1 depth. Non-curative resection is indicated when resected specimens fail to meet the aforementioned criteria or in the presence of: (1) predominantly differentiated intramucosal carcinoma >2 cm in size without ulcers, with undifferentiated components exceeding 2 cm; (2) differentiated superficial submucosal carcinoma ≤3 cm in size, with invasion of undifferentiated components into the submucosa.

### ECura system

The eCura System [8] is comprised of five factors: lymphatic invasion (3 points), invasion depth ≥500μm, tumor diameter > 3 cm, vascular invasion, and positive vertical resection margin (1 point each). Patients were categorized into low-risk (0–1), intermediate-risk (2–4), and high-risk (5–7) groups based on their total score. Each patient in the study was assigned an eCura score and subsequently allocated to the corresponding risk group.

### W-eCura system

The w-eCura system [9] is comprised of five clinical risk factors: lymphatic invasion (3 points), invasion depth ≥1000μm, tumor diameter > 3 cm, vascular invasion, and positive vertical resection margin (1 point each). Patients were categorized into low-risk (0–1), intermediate-risk (2–4), and high-risk (5–7) groups based on their total score. Each patient in the study was assigned a w-eCura score and subsequently placed into the corresponding risk group.

### Statistical analysis

Categorical variables are presented as numbers with percentages, and differences between groups were assessed using Chi-square tests or Fisher’s exact tests. Continuous variables following a normal distribution were reported as Mean±SD, while skewed distributions were described using the median and interquartile range (IQR). Survival analysis was conducted using a Cox proportional hazards regression model. Initially, univariate Cox regression analysis was conducted on all potential independent variables, with statistically significant variables (P < .05) included in the multivariate Cox regression model to calculate Hazard Ratios (HR) and 95% confidence intervals (CI). Survival curves were generated using the Kaplan-Meier method, and differences between curves were assessed using the log-rank test. The Receiver Operating Characteristic (ROC) curve was employed to evaluate and compare the predictive performance of the w-eCura system and eCura system. Logistic regression analysis was utilized for univariate and multivariate analyses to identify risk factors associated with positive outcomes in the intermediate-risk group based on the w-eCura system, with corresponding odds ratios (ORs) and 95% confidence intervals calculated. All analyses were two-tailed, and statistical significance was defined as P < .05. Statistical analyses were performed using R version 4.3 for Windows.

## Results

### Clinicopathological features

This study was conducted among 290 patients with EGC who underwent non-curative resection following ESD to investigate factors associated with non-curative resection (Table 1). The patients had a mean age of 64.42 ± 9.35 years, a median maximum tumor diameter of 4.50 cm, and a median follow-up duration of 32 (15–54) months. No significant differences were observed across gender, lesion location, Paris classification, histological type, presence of ulceration, or vertical resection margin between the CRS-negative group (n = 254) and the CRS-positive group (n = 36) (P > .05). However, significant discrepancies were noted in the w-eCura classification, horizontal resection margin, depth of tumor invasion, and presence of lymphatic and venous invasion between the two groups (P < .05). Specifically, a higher proportion of patients in the CRS-negative group had a lower w-eCura grade compared to the CRS-positive group, whereas a higher proportion of patients in the positive group had a higher w-eCura grade. The CRS-positive group also exhibited a higher prevalence of positive horizontal resection margin, deeper tumor invasion, and lymphatic and venous invasion. These findings suggest that the w-eCura grade, horizontal resection margin, depth of tumor invasion, lymphatic invasion, and venous invasion are crucial factors influencing non-curative resection following ESD.

**Table 1:** Clinicopathological features of patients with post-ESD non-curable resection.

Variables	Total (n = 290)	CRS-negative (n = 254)	CRS-positive (n = 36)	Statistic	*P*
Age, Mean ± SD	64.42 ± 9.35	64.54 ± 9.41	63.56 ± 9.01	t = 0.59	0.554
Size, M (Q₁, Q₃)	4.50 (3.70, 5.60)	4.50 (3.70, 5.50)	4.90 (3.22, 6.00)	Z = −0.05	0.958
Follow-up time, M (Q₁, Q₃)	32.00 (15.00, 54.00)	33.00 (16.00, 56.00)	25.50 (11.25, 48.00)	Z = −1.09	0.276
Gender, n(%)				χ²=0.34	0.561
male	214 (73.79)	186 (73.23)	28 (77.78)		
female	76 (26.21)	68 (26.77)	8 (22.22)		
Location, n(%)				χ²=4.05	0.132
upper1/3 stomach	127 (43.79)	113 (44.49)	14 (38.89)		
middle1/3 stomach	67 (23.10)	54 (21.26)	13 (36.11)		
lower1/3 stomach	96 (33.10)	87 (34.25)	9 (25.00)		
Paris classification, n(%)				χ²=1.12	0.890
I	10 (3.45)	8 (3.15)	2 (5.56)		
IIa	58 (20.00)	52 (20.47)	6 (16.67)		
IIb	133 (45.86)	117 (46.06)	16 (44.44)		
IIc	78 (26.90)	68 (26.77)	10 (27.78)		
III	11 (3.79)	9 (3.54)	2 (5.56)		
w-eCura, n(%)				–	**<.001**
high	3 (1.03)	0 (0.00)	3 (8.33)		
intermediate	65 (22.41)	43 (16.93)	22 (61.11)		
low	222 (76.55)	211 (83.07)	11 (30.56)		
Histological type, n(%)				χ²=0.13	0.717
differentiated types	193 (66.55)	170 (66.93)	23 (63.89)		
undifferentiated types	97 (33.45)	84 (33.07)	13 (36.11)		
Ulceration, n(%)				χ²=0.82	0.366
-	248 (85.52)	219 (86.22)	29 (80.56)		
+	42 (14.48)	35 (13.78)	7 (19.44)		
Horizontal resection margin, n(%)				χ²=10.92	**<.001**
-	205 (70.69)	188 (74.02)	17 (47.22)		
+	85 (29.31)	66 (25.98)	19 (52.78)		
Vertical resection margin, n(%)				χ²=0.83	0.362
-	241 (83.10)	213 (83.86)	28 (77.78)		
+	49 (16.90)	41 (16.14)	8 (22.22)		
Depth of invasion, n(%)				χ²=26.86	**<.001**
M	139 (47.93)	129 (50.79)	10 (27.78)		
SM1	103 (35.52)	89 (35.04)	14 (38.89)		
SM2	41 (14.14)	34 (13.39)	7 (19.44)		
SM3	7 (2.41)	2 (0.79)	5 (13.89)		
Lymphatic invasion, n(%)				χ²=22.91	**<.001**
-	279 (96.21)	250 (98.43)	29 (80.56)		
+	11 (3.79)	4 (1.57)	7 (19.44)		
Venous invasion, n(%)				χ²=10.80	**0.001**
-	264 (91.03)	237 (93.31)	27 (75.00)		
+	26 (8.97)	17 (6.69)	9 (25.00)		

t: t-test, Z: Mann-Whitney test, χ²: Chi-square test, -: Fisher exact, SD: standard deviation, M: Median, Q₁: 1st Quartile, Q₃: 3st Quartile.

### CRS-positive Cox proportional risk model

In this investigation, Cox proportional hazards modeling was employed to analyze factors associated with positive CRS through both univariate and multivariate approaches. The univariate analysis (Table 2) revealed significant correlations (P < .05) between the CRS-positive group and the following factors: tumor location (middle one-third of the stomach), w-eCura grade (intermediate and high), presence of horizontal resection margin involvement, depth of invasion (SM2 and SM3), lymphatic invasion, and venous invasion. Subsequent multifactor analysis (Table 3) independently identified tumor location in the middle one-third of the stomach, w-eCura grade (high and intermediate), and depth of invasion (SM2 and SM3) as influential factors. Patients with tumors in the middle third of the stomach exhibited a higher risk of positive CRS compared to those in the upper one-third of the stomach (HR = 4.56, 95%CI: 1.91–10.88, P < .001). High-grade w-eCura rating conferred the highest risk of positive CRS (HR = 89.91, 95%CI: 11.32–714.45, P < .001), followed by intermediate grade (HR = 11.12, 95%CI: 3.50–35.30, P < .001). Patients with invasion depths of SM2 (HR = 3.07, 95%CI: 1.07–8.83, P = .037) and SM3 (HR = 9.99, 95%CI: 2.79–35.83, P < .001) had significantly elevated risks of CRS-positive classification compared to those with mucosal invasion. These findings underscore the significance of tumor location, w-eCura grade, and depth of invasion as independent determinants of positive CRS outcomes.

**Table 2:** Single-factor COX proportional risk model.

Variables	β	S.E	Z	*P*	HR (95%CI)
Gender					
female					1.00 (Reference)
male	0.19	0.40	0.47	0.638	1.21 (0.55 ~ 2.65)
Location					
upper1/3 stomach					1.00 (Reference)
middle1/3 stomach	0.89	0.39	2.29	**0.022**	2.44 (1.14 ~ 5.21)
lower1/3 stomach	0.07	0.43	0.16	0.871	1.07 (0.46 ~ 2.48)
Paris classification					
I					1.00 (Reference)
IIa	−0.53	0.82	−0.65	0.517	0.59 (0.12 ~ 2.92)
IIb	−0.66	0.75	−0.88	0.381	0.52 (0.12 ~ 2.26)
IIc	−0.23	0.78	−0.30	0.767	0.79 (0.17 ~ 3.64)
III	0.00	1.00	0.00	0.998	1.00 (0.14 ~ 7.15)
w-eCura					
low					1.00 (Reference)
intermediate	2.20	0.37	5.94	**<.001**	9.00 (4.36 ~ 18.60)
high	3.28	0.66	4.97	**<.001**	26.51 (7.27 ~ 96.60)
Histological type					
differentiated types					1.00 (Reference)
undifferentiated types	0.13	0.35	0.37	0.709	1.14 (0.58 ~ 2.25)
Ulceration					
-					1.00 (Reference)
+	0.62	0.43	1.46	0.144	1.86 (0.81 ~ 4.28)
Horizontal resection margin					
-					1.00 (Reference)
+	1.00	0.33	2.98	**0.003**	2.71 (1.41 ~ 5.21)
Vertical resection margin					
-					1.00 (Reference)
+	0.08	0.40	0.21	0.836	1.09 (0.49 ~ 2.39)
Depth of invasion					
M					1.00 (Reference)
SM1	0.52	0.41	1.26	0.206	1.69 (0.75 ~ 3.81)
SM2	1.28	0.50	2.57	**0.010**	3.58 (1.35 ~ 9.47)
SM3	2.81	0.55	5.09	**<.001**	16.62 (5.63 ~ 49.03)
Lymphatic invasion					
-					1.00 (Reference)
+	1.90	0.42	4.51	**<.001**	6.69 (2.93 ~ 15.29)
Venous invasion					
-					1.00 (Reference)
+	1.39	0.39	3.59	**<.001**	4.00 (1.88 ~ 8.51)

HR: Hazard Ratio, CI: Confidence Interval.

**Table 3:** Multi-factor COX proportional risk model.

Variables	β	S.E	Z	*P*	HR (95%CI)
Location					
upper1/3 stomach					1.00 (Reference)
middle1/3 stomach	1.52	0.44	3.43	**<.001**	4.56 (1.91 ~ 10.88)
lower1/3 stomach	0.55	0.45	1.23	0.220	1.74 (0.72 ~ 4.22)
w-eCura					
low					1.00 (Reference)
high	4.50	1.06	4.25	**<.001**	89.91 (11.32 ~ 714.45)
intermediate	2.41	0.59	4.09	**<.001**	11.12 (3.50 ~ 35.30)
Horizontal resection margin					
-					1.00 (Reference)
+	−0.90	0.50	−1.81	0.071	0.41 (0.15 ~ 1.08)
Depth of invasion					
M					1.00 (Reference)
SM1	0.24	0.46	0.52	0.604	1.27 (0.52 ~ 3.12)
SM2	1.12	0.54	2.08	**0.037**	3.07 (1.07 ~ 8.83)
SM3	2.30	0.65	3.53	**<.001**	9.99 (2.79 ~ 35.83)
Lymphatic invasion					
-					1.00 (Reference)
+	−0.51	0.60	−0.85	0.397	0.60 (0.18 ~ 1.96)
Venous invasion					
-					1.00 (Reference)
+	0.54	0.55	0.97	0.330	1.71 (0.58 ~ 5.02)

HR: Hazard Ratio, CI: Confidence Interval.

### Effects of eCura and w-eCura grades on survival time

**Fig 1:**
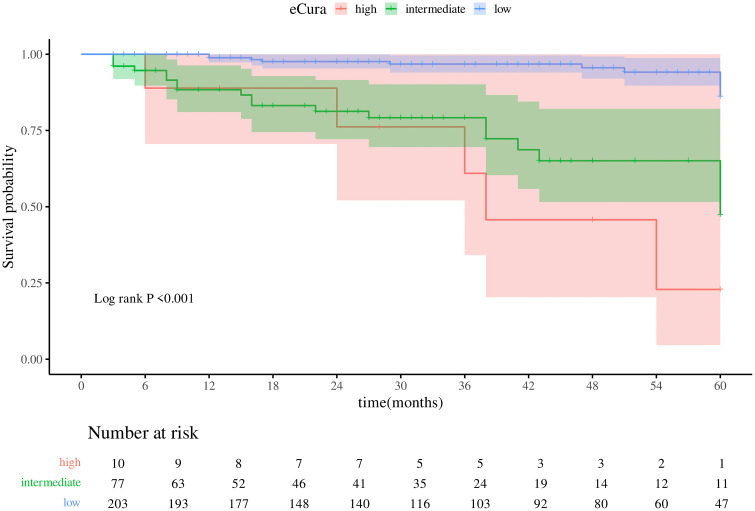
Kaplan-Meier survival curve of eCura.

**Fig 2:**
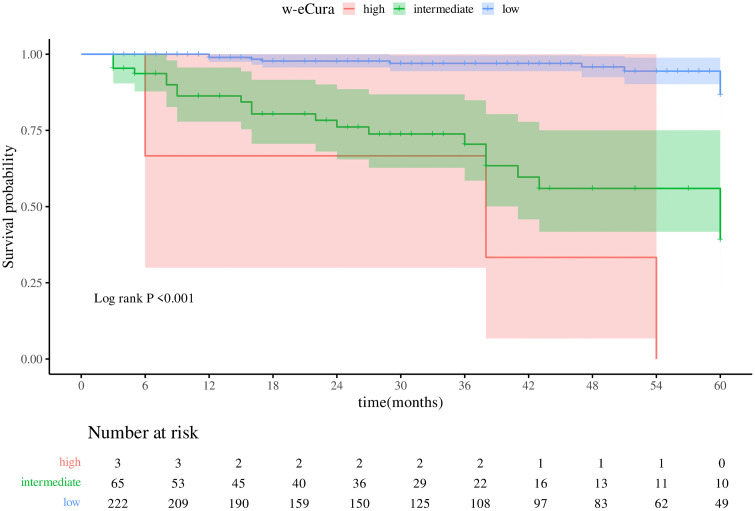
Kaplan-Meier survival curve of w-eCura.

In this investigation, the Kaplan-Meier survival curve was employed to assess the impact of eCura and w-eCura grading on patient survival duration. The analysis revealed a significant association between both grading systems and survival time (Logrank P < .001). Higher eCura and w-eCura grades were linked to shorter median survival times and increased event frequencies. Specifically, patients with elevated eCura ratings exhibited a median survival time of 38 months and an event rate of 1666.67/1000 person-months (Fig 1; Table 4). Similarly, individuals with high w-eCura grades had a median survival time of 38 months, but with a lower event rate of 500.00/1000 person-months (Fig 2; Table 5). These findings suggest that while both grading methodologies effectively predict survival outcomes, w-eCura grading may offer enhanced sensitivity in identifying high-risk patients promptly.

**Table 4:** eCura Median Schedule.

Variables	N	Events	Median (95%CI)	Rate/1000 (person- months)	*Logrank P value*
eCura					**<.001**
high	10	5	38.00 (36.00 – NA)	1666.67	
intermediate	77	20	60.00 (60.00 – NA)	6666.67	
low	203	11	NA (NA – NA)	2750.00	

**Table 5:** w-eCura median schedule.

Variables	N	Events	Median (95%CI)	Rate/1000 (person-months)	*Logrank P value*
w-eCura					**<.001**
high	3	3	38.00 (6.00 – NA)	500.00	
intermediate	65	22	60.00 (41.00 – NA)	7333.33	
low	222	11	NA (NA – NA)	3666.67	

### Comparison of the predictive ability of eCura and w-eCura models for CRS-positive classification

**Fig 3:**
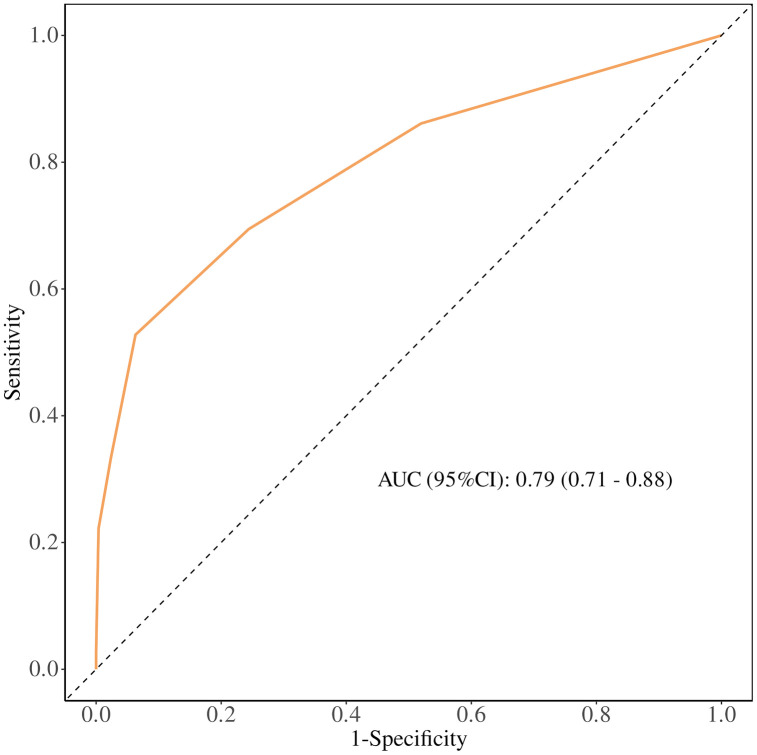
ROC curve of eCura model.

**Fig 4:**
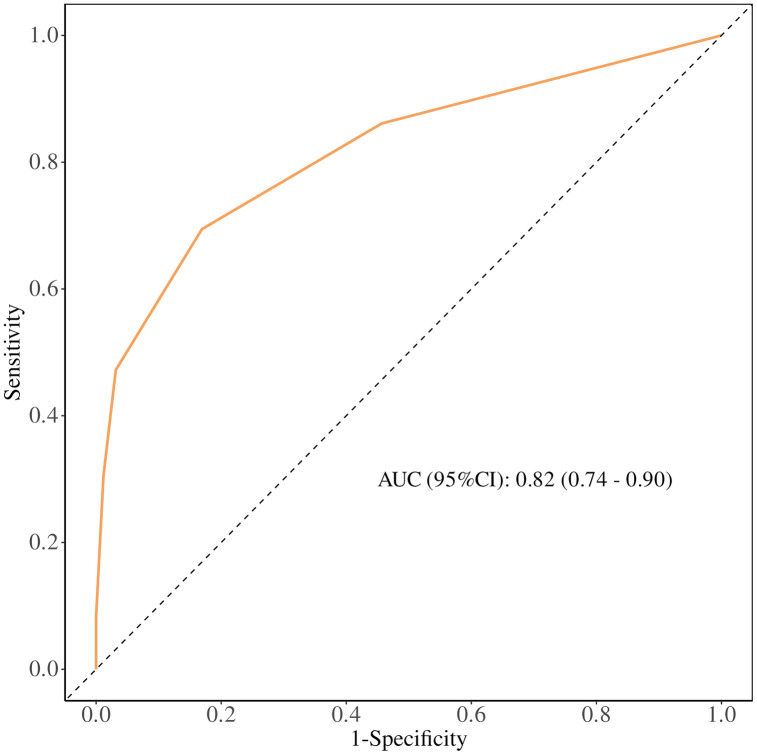
ROC curve of w-eCura model.

The predictive performance of the eCura and w-eCura models for CRS-positive classification was assessed using ROC curve analysis. The AUC for the w-eCura model was.82 (95%CI:.74−.90) (Fig 3), surpassing the AUC of the eCura model at.79 (95%CI:.71−.88) (Fig 4). These findings indicate that the w-eCura model exhibits superior accuracy in forecasting CRS-positive outcomes, enabling enhanced differentiation between high-risk and low-risk patients and offering a more dependable basis for clinical decision-making. This outcome underscores the effectiveness and practicality of the w-eCura model in evaluating CRS risk among patients, demonstrating its heightened predictive capacity for CRS-positive nodes compared to the eCura model.

### Analysis of factors influencing positive CRS classification in w-eCura intermediate-risk patients

In this investigation, logistic regression analysis was conducted to identify factors influencing the occurrence of positive CRS in the intermediate risk w-eCura subgroup (Table 6). Univariate analysis revealed significant correlations (P < .05) between positive CRS outcome and vertical resection margin, horizontal resection margin, and infiltration depth, while variables such as gender, location, Paris classification, histological type, ulcer presence, lymphatic involvement, and venous infiltration showed no significant correlation. Subsequent multifactorial analysis confirmed that vertical resection margin and infiltration depth (SM3) were independent influencing factors. Patients with positive vertical resection margins exhibited a substantially elevated risk (OR=116.22, 95%CI: 3.06–4411.47, P = .010), and those with SM3 infiltration depth similarly displayed a significantly higher risk of CRS-positive classification compared to those with the M layer (OR=163.75, 95%CI: 2.29–11684.78, P = .019). These findings underscore the critical roles of resection margin status and infiltration depth as autonomous factors influencing CRS-positivity in the intermediate risk cohort defined by the w-eCura system.

**Table 6:** Logistic regression analysis of influencing factors for positive CRS in w-eCura intermediate-risk group.

Variables	Univariate analysis					Multifactorial analysis				
	β	S.E	Z	*P*	OR (95%CI)	β	S.E	Z	*P*	OR (95%CI)
Gender										
male					1.00 (Reference)					1.00 (Reference)
female	−1.23	0.82	−1.51	0.132	0.29 (0.06 ~ 1.45)	−1.85	1.43	−1.29	0.196	0.16 (0.01 ~ 2.59)
Location										
upper1/3 stomach					1.00 (Reference)					1.00 (Reference)
lower1/3 stomach	0.12	0.64	0.18	0.854	1.12 (0.32 ~ 3.95)	0.70	1.03	0.68	0.495	2.02 (0.27 ~ 15.26)
middle1/3 stomach	0.97	0.65	1.48	0.138	2.62 (0.73 ~ 9.39)	2.60	1.39	1.87	0.061	13.49 (0.89 ~ 204.91)
Paris classification										
I					1.00 (Reference)					1.00 (Reference)
IIa	−0.85	1.38	−0.61	0.539	0.43 (0.03 ~ 6.41)	−3.78	2.36	−1.60	0.110	0.02 (0.00 ~ 2.35)
IIb	0.12	1.29	0.09	0.927	1.12 (0.09 ~ 14.20)	−1.90	2.13	−0.90	0.371	0.15 (0.00 ~ 9.62)
IIc	0.58	1.32	0.44	0.662	1.78 (0.13 ~ 23.52)	−1.39	2.22	−0.63	0.532	0.25 (0.00 ~ 19.46)
III	0.00	1.73	0.00	1.000	1.00 (0.03 ~ 29.81)	−4.37	3.18	−1.38	0.169	0.01 (0.00 ~ 6.38)
Histological type										
differentiated types					1.00 (Reference)					1.00 (Reference)
undifferentiated types	0.70	0.56	1.26	0.209	2.01 (0.68 ~ 6.00)	1.56	0.86	1.80	0.072	4.75 (0.87 ~ 25.84)
Ulceration										
-					1.00 (Reference)					1.00 (Reference)
+	0.29	0.71	0.41	0.684	1.33 (0.33 ~ 5.33)	1.42	1.21	1.17	0.241	4.12 (0.39 ~ 44.08)
Horizontal resection margin										
-					1.00 (Reference)					1.00 (Reference)
+	−1.66	0.64	−2.58	**0.010**	0.19 (0.05 ~ 0.67)	−1.27	1.25	−1.02	0.309	0.28 (0.02 ~ 3.24)
Vertical resection margin										
-					1.00 (Reference)					1.00 (Reference)
+	1.80	0.88	2.03	**0.042**	6.03 (1.06 ~ 34.17)	4.76	1.86	2.56	**0.010**	116.22 (3.06 ~ 4411.47)
Depth of invasion										
M					1.00 (Reference)					1.00 (Reference)
SM1	1.20	0.74	1.63	0.104	3.33 (0.78 ~ 14.14)	1.38	1.21	1.15	0.252	3.99 (0.37 ~ 42.65)
SM2	0.94	0.72	1.30	0.194	2.56 (0.62 ~ 10.55)	1.77	1.29	1.38	0.169	5.88 (0.47 ~ 73.42)
SM3	2.94	1.25	2.36	**0.018**	19.00 (1.65 ~ 218.47)	5.10	2.18	2.34	**0.019**	163.75 (2.29 ~ 11684.78)
Lymphatic invasion										
-					1.00 (Reference)					1.00 (Reference)
+	1.05	0.73	1.44	0.150	2.87 (0.68 ~ 12.02)	1.08	1.43	0.75	0.452	2.93 (0.18 ~ 48.38)
Venous invasion										
-					1.00 (Reference)					1.00 (Reference)
+	0.51	0.56	0.90	0.368	1.66 (0.55 ~ 5.03)	1.01	1.12	0.90	0.369	2.74 (0.30 ~ 24.57)

## Discussion

Gastric cancer, characterized by its high incidence and mortality rates, is particularly prevalent in countries like China [1], remaining a significant public health focus. Early detection, diagnosis, and treatment are crucial for improving outcomes, as the prognosis for early-stage gastric cancer is notably more favorable compared to advanced stages. While radical gastrectomy with lymph node dissection has historically been the primary treatment for EGC [10], the strategy is associated with increased trauma and mortality risk. However, technological advancements have led to ESD emerging as the preferred treatment for EGC due to its lower risk of LNM. ESD enables complete tumor resection while preserving stomach anatomy, thereby reducing the likelihood of postoperative dysfunction [14]. Clinical studies have consistently demonstrated that the long-term prognosis following ESD is comparable to that of surgical intervention [15,16].

Several studies have indicated that despite EGC being limited to the mucosal layer, the incidence of LNM ranges from 2.2% to 4.57% [5,17]. Despite advancements in preoperative diagnostic technologies, accurately assessing LNM remains challenging, necessitating postoperative pathology for precise evaluation. ESD is a local treatment modality for EGC but falls short of achieving complete radical resection due to its inability to sample or clear lymph nodes. The identification of risk factors for non-curative outcomes post-ESD, such as LNM or local residual cancer, remains incomplete. Nonetheless, the prevalence of lymph node metastases in patients with non-curative EGC following ESD and subsequent surgical intervention is relatively low, ranging from 6.3% to 12.7% [18–20]. While the survival rates following resection of non-curative EGC have improved, there exists a concern of potential overtreatment [21]. Implementing additional surgical interventions for all non-curative EGC cases post-ESD may lead to unnecessary surgeries in over 90% of patients. Radical surgery for EGC poses a higher risk of complications compared to ESD, particularly in older patients or those with comorbidities, thereby increasing the associated mortality risks.

Identifying patients with EGC who have a low or negligible risk of LNM following non-curative resection is essential to prevent unnecessary surgeries. This can significantly impact patients’ quality of life by avoiding surgically-associated risks in favor of close monitoring. Therefore, accurately pinpointing the high-risk subgroup necessitating salvage surgery post non-curative ESD is paramount for optimal patient management.

This study investigated the risk factors for positive CRS after non-curable ESD in patients with EGC. The analysis revealed that factors such as gender, Paris classification, pathological type, and presence of ulcer did not significantly impact positive CRS. Single-factor Cox analysis identified middle 1/3 location, SM2/SM3 invasion depth, positive vertical resection margin, lymphatic invasion, venous invasion, and intermediate to high risk w-eCura groups as significant risk factors for positive CRS. Subsequent multiple-factor Cox analysis demonstrated that EGCs located in the middle 1/3 of the stomach and SM2/SM3 invasion depth were independent risk factors for positive CRS. Prior research has presented conflicting findings on the role of lesion location as a standalone risk factor for LNM in patients with EGC who have not undergone curative resection [22,23]. While some studies have indicated that lesion location does not independently predict LNM, others have linked tumor localization in the gastric antrum with a higher likelihood of lymph node involvement [24]. This result is inconsistent with the finding of the present study, which demonstrated that compared to the upper 1/3, patients with EGCs in the middle 1/3 stomach were more likely to experience CRS positive classification. This discrepancy may be attributed to the broader definition of CRS utilized in our study, encompassing not only LNM but also residual tumor and recurrence. The inherent anatomical challenges in operating on the middle third of the stomach, particularly in ESD, may lead to higher rates of residual disease, thus increasing the likelihood of CRS positivity. Regarding the influence of depth of invasion on CRS in patients with non-curatively resected EGC, our investigation revealed that CRS became a significant risk factor necessitating further surgical intervention when invasion depth extended to SM2 and SM3 layers. Consistent with prior evidence linking deeper invasion (SM2 or beyond) to LNM [25], our study aligns with these findings, underscoring the association between deeper invasion depth and the need for additional surgical measures.

This study assessed the risk stratification in patients with EGC who underwent non-curative. The findings indicated that patients classified in the intermediate and high-risk groups were independently associated with a higher risk of curative resection failure in non-curable EGC cases. Hence, the w-eCura score emerged as a significant predictor of curative resection success. The w-eCura score represents an enhanced version of the eCura system introduced by European researchers in recent years. Originally conceived by Hatta et al. [8], the eCura system was designed to predict LNM and comprises five parameters: lymphatic invasion, tumor size, positive vertical margin, venous invasion, and submucosal invasion. Hatta et al. also demonstrated the utility of this system in guiding decisions regarding salvage surgery post non-curative ESD [26]. The w-eCura score, based on the eCura system, utilizes a submucosal invasion depth criterion of ≥1 mm, in contrast to the ≥ 0.5 mm criterion in the eCura system. It categorizes patients into three risk groups: low-risk (0–1), intermediate-risk (2–4), and high-risk (5–7). Validation studies have demonstrated that the w-eCura score exhibits superior discriminatory capacity, indicating its enhanced discriminatory performance compared to the eCura system specifically within European populations.

In this investigation, we conducted a comparative analysis of the predictive diagnostic efficacy of the eCura score and the w-eCura score concerning the detection of CRS-positive classification and the estimation of survival duration. Survival curve analysis revealed significant impacts of different risk categories within the eCura score and the w-eCura score on survival duration, with the w-eCura score demonstrating improved survival prognostication for patients classified as low and intermediate risk. Furthermore, assessment of the AUC of the ROC curve indicated the clinical utility of both the w-eCura score and the eCura score in prognosticating the CRS positive group, with the w-eCura score exhibiting enhanced predictive accuracy compared to the eCura score in this context (AUC: 0.82 vs. 0.79).These findings suggest that the w-eCura score may serve as a more suitable indicator for evaluating the necessity of salvage surgery following non-curative ESD in cases of EGC. However, given the limitations of a small sample size and a single-center study design, further validation studies are warranted.

The eCura scores exhibited a robust correlation with cancer-related outcomes among patients with non-curable ESD. High-risk populations benefited from salvage surgery, while low-risk groups were well managed with follow-up alone. Conversely, the optimal treatment approach for the intermediate cohort remained uncertain [26]. This investigation found a significant association between the high-risk subset of w-eCura and CRS classification, in contrast to the low-risk subset of w-eCura, which aligned with earlier studies advocating for follow-up without supplementary surgery in low-risk cases [9]. Nonetheless, the necessity of additional surgery for patients classified as intermediate risk by the w-eCura score remains a subject of debate. We conducted an analysis to investigate factors influencing incomplete resection in patients with EGC who underwent non-curative resection with a moderate w-eCura score. Our aim was to identify key factors guiding precise treatment strategies. Our findings indicate that a positive margin and invasion depth of SM3 were autonomous risk factors for CRS in patients with non-curative ESD at intermediate risk, according to the w-eCura scale. The impact of a positive margin on the prognosis following non-curative resection of EGC remains debated. A meta-analysis involving 1,720 EGC patients revealed a significant association between positive vertical resection margin (p < 0.001) and LNM, while horizontal resection margin was not linked to this risk. Prior research has highlighted the importance of both horizontal and vertical resection margins in local recurrence risk [27]. The present study suggests that a positive horizontal resection margin may influence CRS, with a positive vertical resection margin being an independent risk factor for CRS-positive classification. Prior studies have indicated that there are no significant differences in LNM and cancer recurrence rates between SM1 and SM2 invasive cancers [28]. Therefore, for SM3 invasive cancers, salvage surgery is recommended. Conversely, for SM1 or SM2 invasive cancers with negative ESD margins and no other risk factors, close monitoring may be sufficient, especially for elderly patients with comorbidities. Prospective, large scale, multi-center studies with robust sample sizes are required to validate the findings described herein.

Additionally, the relatively small number of patients in the w-eCura high-risk group (n = 3) resulted in wide confidence intervals in the logistic regression analysis, which may reduce the precision of the estimates and affect the stability of the model. Therefore, our conclusions, particularly those regarding the high-risk population, warrant validation in larger, multicenter prospective cohorts.

## Conclusion

1. The w-eCura score is effective in assessing the need for salvage surgery following non-curative ESD in EGC, demonstrating superior accuracy compared to the eCura model.
2. For patients with non-curative ESD and a moderate risk w-eCura score, particularly those with positive vertical resection margin or SM3 invasion depth, proactive consideration of salvage surgery is advised.
